# Progression of Enamel Demineralisation Around Fissure Sealants: Optical Coherence Tomography Study

**DOI:** 10.3290/j.ohpd.b2448625

**Published:** 2021-12-18

**Authors:** Alaa Turkistani, Turki A. Bakhsh

**Affiliations:** a Assistant Professor, Department of Restorative Dentistry, Faculty of Dentistry, King Abdulaziz University, Saudi Arabia. Study design; data acquisition, analysis and interpretation; drafted and critically revised the manuscript.; b Associate Professor, Department of Restorative Dentistry, Faculty of Dentistry, King Abdulaziz University, Saudi Arabia. Study concept; data interpretation; consulted on and performed statistical evaluation; critically revised the manuscript.

**Keywords:** enamel, fissure sealant, fluoride, optical coherence tomography, tooth demineralisation

## Abstract

**Purpose:**

To evaluate the progression of enamel demineralisation around fissure sealants using cross-polarisation optical coherence tomography (CP-OCT).

**Materials and Methods:**

Three fissure sealants; Fuji Triage (FJ, GC), Beautisealant (BT, Shofu) and Helioseal resin sealant (HL, Ivoclar Vivadent) were placed in cavities (3 × 0.5 × 1 mm) prepared in bovine enamel blocks (n = 5). After 7-day artificial saliva incubation, specimens were subjected to demineralisation for 4 weeks (pH 4.5). CP-OCT scans (Santec) were acquired for each specimen after 1, 2 and 4 weeks of demineralisation and lesions were quantitatively measured to analyse lesion progression.

**Results:**

Repeated-measures ANOVA demonstrated that lesion size was statistically significantly affected by demineralisation period, fissure sealant type, and their interaction (*P* < 0.001). At 4 weeks, the highest mean value of lesion size was observed in HL group while FJ group showed the lowest. The rate of lesion progression was slower in FJ and statistically significantly different from BT (*P* < 0.05), which in turn was statistically significantly different than HL (*P* < 0.001).

**Conclusion:**

Fissure sealants that actively release ions are capable of improving the acid resistance of adjacent enamel. Beautisealant showed increased demineralisation inhibition compared to conventional resin sealant, but less than that provided by glass-ionomer sealant.

Caries arises as a consequence of imbalance in the equilibrium between demineralisation and remineralisation at the interface between biofilm and tooth surface.^1, 6^ When a progressive interaction with acids is not stopped or reversed, dissolution of the surface crystallites may result, which can progress into localised destruction or cavitation.^6^ This process may occur on any tooth surface where a mature biofilm is established.^1, 6^ However, occlusal surface of posterior teeth is more susceptible to caries as plaque accumulates on the retentive grooves exposing the surface to repeated acid attack.^1, 6, 15^ In fact, early onset and high prevalence of occlusal caries in recently erupted molars with limited mechanical function were reported.^1, 6^ Furthermore, severe lesions are most notable in paediatric patients, particularly in high-risk children.^16^

Currently, caries management is based on a medical model that embraces risk-based strategies aiming to arrest disease progression by altering the cariogenic environment, treat established lesions with microinvasive intervention and prevent new lesions.^10, 30^ Such prevention-focused concept implements measures that emphasise on isolation of the surface, increasing the resistance of tooth structure and remineralisation of early lesions.^30^ Sealants have been effectively used as a preventive intervention to fissure caries in high-risk individuals.^1, 29^ The hermetic sealing of the fissure creates a barrier to acid ingression, inhibiting caries development.^14^ Fissure sealants have been also suggested for controlling initial caries lesion and arresting its progression on occlusal and proximal surfaces as well.^10, 16^ In addition to physical protection, sealants with ion-releasing property seem to provide cariostatic effect on adjacent tooth structure.^5^

Glass-ionomer materials are acknowledged for their desirable preventive effects through the release of fluoride over prolonged period, reinforcing hard tissue structure.^9^ Lately, a hybrid material that uses a resin base and surface pre-reacted glass-ionomer (S-PRG) fillers called ‘giomer’ is introduced. It combines the inhibitory effect of glass-ionomer such as fluoride release and recharge with the properties of resin composite, such as superior aesthetics, improved physical properties, easy polishability and smooth surface finish.^11^ Although the superior inhibitory effect of glass ionomers to conventional resin sealants has been widely reported,^5, 29^ the effect of giomers has yet to be investigated. In addition, clinical studies comparing inhibitory effect of fluoridated to non-fluoridated sealants exhibited contradictory results.^1, 14, 16^ A previous study suggested that glass-ionomer and resin sealants had comparable preventive effects in children.^8^ Another study showed no statistically significant difference in mineral loss between incipient lesions sealed with fluoridated sealants and those sealed with conventional sealants.^27^

Another key strategy in modern caries management is early detection.^30^ However, assessment of lesion severity and activity based on the visual and tactile criteria is challenging.^18^ Besides, clinical monitoring of sealant margins for secondary caries is crucial for minimal intervention.^30^ Optical coherence tomography (OCT) has emerged as a non-invasive objective diagnostic tool that can provide real-time cross-sectional images of internal structures non-destructively.^18, 22^ Cross-polarisation optical coherence tomography (CP-OCT) is a variant of OCT in which the backscattered light is detected with oscillation level perpendicular to linearly polarised signals.^2, 3, 7^ CP-OCT has exhibited high sensitivity and specificity for early detection of carious lesions with improved image resolution.^3, 7^

The objective of this laboratory study is to monitor lesion progression around enamel margins of fissure sealants using CP-OCT, aiming to investigate the effect of sealants on demineralisation progression rate. The null hypotheses tested were: (1) there is no difference in marginal lesion size among the tested groups at different demineralisation periods; (2) there is no correlation between sealant type and demineralisation period on lesion size.

## Materials and Methods

### Specimens Preparation

Freshly extracted bovine incisors were collected after obtaining the approval of King Abdulaziz University’s Research Ethical Committee and following the Declaration of Helsinki. Enamel blocks were sectioned from the crown and embedded in resin. The surface of each specimen was polished with silicone papers (SiC) to attain a flat surface. Cavities, 3 mm long × 0.5 mm wide × 1 mm deep, were prepared using high-speed diamond burs (Meisinger, Centennial, CO, USA). A new bur was used for every five cavities and all cavities were observed under OCT to confirm standard dimensions. Specimens were categorised into three groups of five specimens each. In the HL group, conventional resin sealant (Helioseal, Ivoclar/Vivadent, Schaan, Liechtenstein) was used to fill the cavities. In the FJ group, cavities were filled with Fuji Triage (FJ, GC, Tokyo, Japan), while Beautisealant (Shofu, Kyoto, Japan) was used in the BT group. An LED light-cure unit with 1,200 mW/cm^2^ output power intensity was used to cure the resin sealants (3M Oral Care Elipar S10, St. Paul, MN, USA). Each material was applied according to manufacturers’ recommendations as explained in Table 1.

**Table 1:** Materials used in the study

Material (Abbreviation) Manufacturer	Chemical composition	Application method
Helioseal (HL) Ivoclar Vivadent	Bis-GMA, TEGDMA, titanium dioxide, stabilisers and catalysts	Apply etchant for 30 s, rinse thoroughly and dry, apply the sealant and wait for 15 s. Light-cure for 20 s
Beautisealant (BT) Shofu	Primer: acetone, distilled water, carboxylic acid monomer, phosphonic acid monomer and others Paste: S-PRG fillers, UDMA, TEGDMA, microfumed silica and others	Apply primer, leave undisturbed for 5 s, air dry gently for 5 s Apply the paste and light-cure for 10 s
Fuji Triage (FJ) GC Corporation	Aluminofluorosilicate glass, polyacrylic acid, distilled water, pigment, polybase carboxylic acid	Mix the capsule 10 s at high speed, load into the applier, extrude the mixture and use a brush to spread a thin film

Abbreviations: Bis-GMA: bisphenol A-glycidyl methacrylate; TEGDMA: triethyleneglycol dimethacrylate; S-PRG: surface pre-reacted glass; UDMA: urethane dimethacrylate.

After 24 h, the surface of each specimen was polished using 2,000-grit SiC paper to ensure the absence of any excess material covering the surface. Then each specimen was immersed in 1 ml of artificial saliva (pH 6.5) for 7 days.^24–26^ Two layers of varnish were applied to the specimen’s surface, leaving a 0.5 mm-wide window of exposed enamel surrounding the margins. Then, each specimen was separately immersed in 1 ml of demineralising solution (0.9 mm KH_2_PO_4_, 1.5 mm CaCl_2_, 50 mm CH_3_COOH, 0.02% NaN_3_; pH 4.5) and placed in an incubator at 37°C.^24–26^ Solution was refreshed daily and specimens were washed with deionised water and blotted dry with tissue paper before reimmersion.

### OCT Imaging

The CP-OCT system (IVS-300, Santec, Komaki, Japan) utilised in this study constructs images with a central wavelength of 1,330 nm, optical resolution in air of 12 μm in depth and 30 μm in width and a sweep rate of 30 kHz power, as within American National Standard Institute safe ranges. For each specimen, serial 2D images along the x- and y-axes were acquired at 250 µm intervals after 1, 2 and 4 weeks of demineralisation. At each time point, the specimen was rinsed and blotted by tissue keeping the surface moist. A handheld scanning probe was placed 1 mm from the specimen positioned on a micrometer platform, with the beam oriented perpendicular to the surface.

To evaluate the progress of demineralised lesion, raw OCT data (500 × 925 pixels corresponding to 5 mm × 8.2 mm for each 2D image) were imported to image analysis software (ImageJ, National Institute of Health, Bethesda, MD, USA) and cavitations formed due to demineralisation were outlined and cross-sectional areas of tissue loss were measured in mm^2^.

### Statistical Analysis

To compare the lesion size among the groups, ANOVA with repeated measures and multiple comparisons with Bonferroni corrections were performed at a statistical significance level of 0.05 (SPSS, Chicago, IL, USA).

## Results

**Fig 1:**
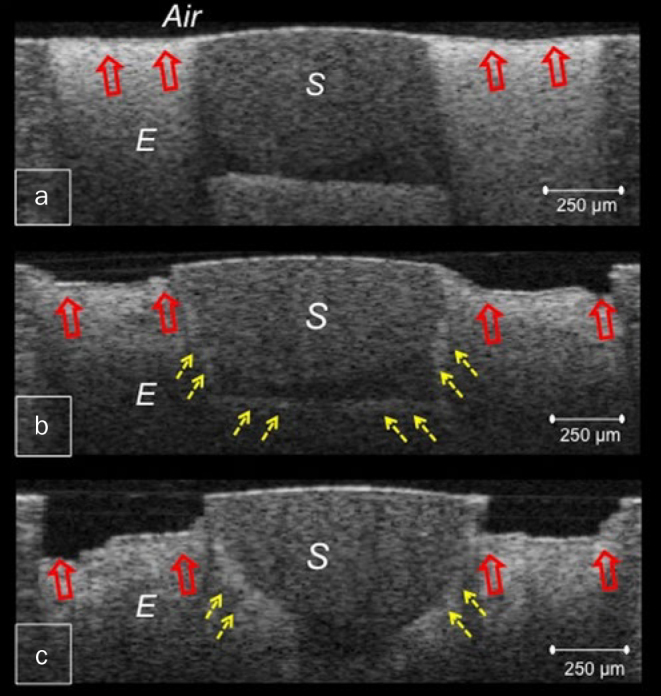
CP-OCT images obtained from HL sealant after 1 w (a), 2 w (b) and 4 w (c). Blank arrows indicate surface demineralisation that progressed into deep cavitation. Broken arrows show lesion progression along the inner wall of the sealant. E: enamel, S: sealant.

**Fig 2:**
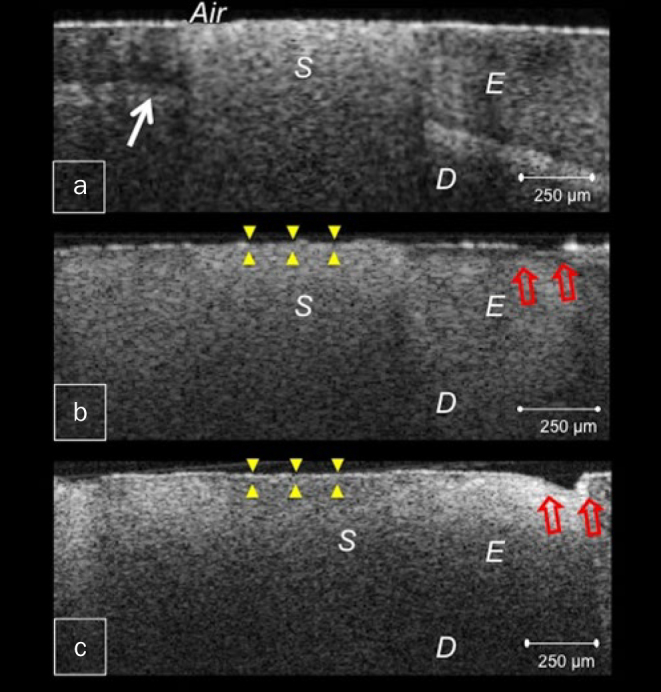
CP-OCT images from BT specimen. (a) Bright clusters of demineralised lesions at a distance from the margin after 1 w. Arrow denotes dentinoenamel junction (DEJ); (b) After 2 w, higher reflectivity surrounding the sealant indicates demineralisation of the entire enamel substrate with surface breakdown away from the margin (blank arrows), progressing into cavitation after 4 w (c), sealant surface was slightly irregular (triangles) with strong backscattered reflection (c). D: dentine, E: enamel, S: sealant.

**Fig 3:**
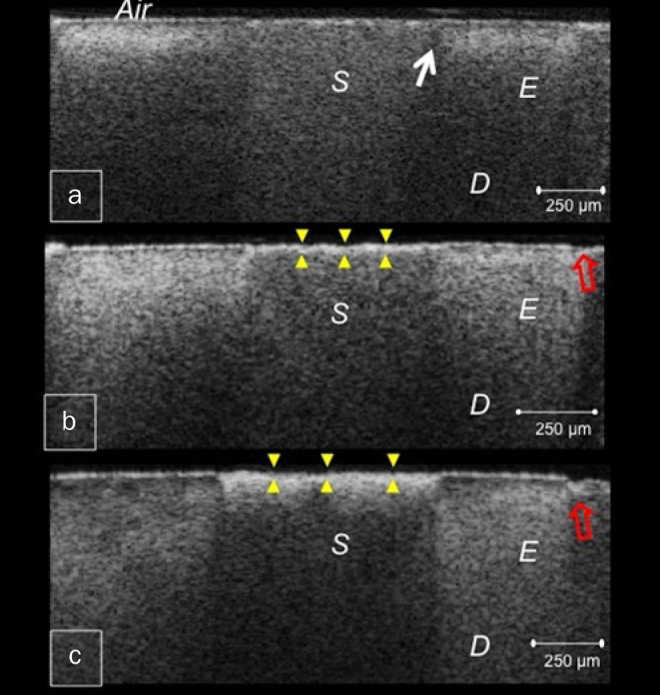
OCT B-scans of FJ. (a) B-scan after 1 w of demineralisation showing subsurface lesion with sound enamel adjacent to the sealant margin (arrow); (b) and (c) surface remained intact despite the lesion progression in the successive weeks. Blank arrows indicate shallow enamel breakdown at this margin. Triangles denote surface irregularities. D: dentine, E: enamel, S: sealant.

**Fig 4:**
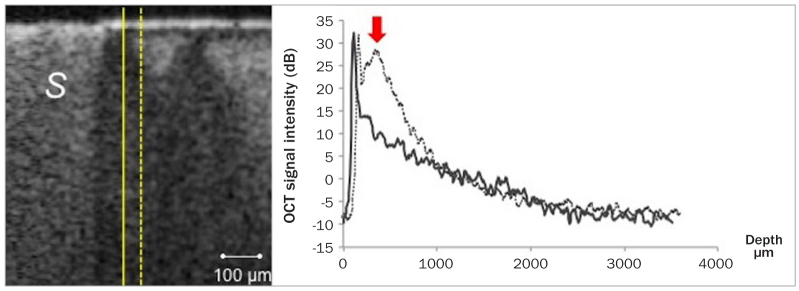
A-scan (OCT signal intensity) profiles plotted on the same cross-section at different distances from the margin of FJ sealant. No evident change in signal intensity can be detected in solid line A-scan, indicating intact enamel adjacent to the sealant. The dashed line A-scan shows high intensity in backscatter signal (arrow) induced by demineralised enamel. S: sealant.

**Fig 5:**
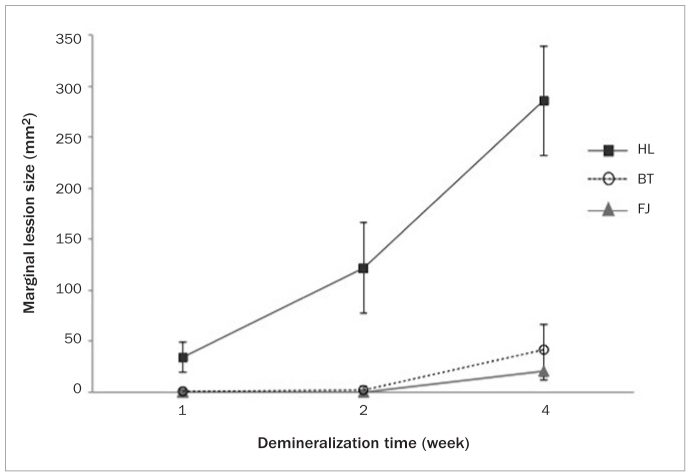
Mean lesion size of the tested groups across the demineralisation period.

Representative CP-OCT images are shown in Figures 1, 2, 3 and 4. Mean lesion size was significantly affected by demineralisation period and fissure sealant type (*P* < 0.001), which had a statistically significant interaction effect (*P* < 0.001). Of the three fissure sealants in the present study, FJ group demonstrated the lowest mean value with a slow progression rate, while HL group showed the highest (Fig 5). Repeated-measures ANOVA test showed that lesion progression in FJ and BT was significantly different from HL (*P* < 0.001). The difference between FJ and BT was also statistically significant (*P* < 0.05).

The OCT images showed that both FJ and BT had less demineralisation than the resin sealant HL. Enamel around HL demonstrated cavitated lesions only after 1 week of demineralisation, appearing as dark areas of tissue loss with underlying increased signal intensity of demineralised enamel, and progressing into deep cavitations in the successive weeks (Fig 1). Whereas in FJ and BT, bright zones of subsurface lesions of increasing depth and severity were produced, which advanced into enamel breakdown and shallow cavitations (Figs 2 and 3). These cavitations were formed at the lesion periphery, preserving enamel surface near the sealant margin (Figs 2c and 3c).

In some cross-sections, intact enamel resisting demineralisation was observed at margins of FJ and BT with subsurface lesion at a distance from the margin, as indicated by the A-scan profiles in Figure 4. On the other hand, no inhibition zones were demonstrated around the fissure sealant HL.

## Discussion

The long-term clinical effectiveness of fissure sealant relies on its ability to achieve high-quality adhesion to enamel and inhibit marginal demineralisation.^5, 21^ This study examined demineralisation progression around three types of fissure sealants, glass-ionomer sealant, resin-based sealant containing S-PRG fillers bonded by self-etching primer and conventional resin sealant bonded by enamel etching. The method used in this study tests the materials’ demineralisation inhibition properties by inducing the formation of cavitated lesions for objective comparison between the materials.^24–26^ Bovine enamel was used as a substitute to human enamel as it has comparable physicochemical features but more consistent composition and mineral content, promoting less variable results.^17^

In this study, OCT imaging was adopted as a non-destructive tool for objective monitoring of demineralisation progression. The demineralisation process develops numerous enamel porosities, causing depolarisation and scattering of the incident light with increase in reflectivity. In the corresponding OCT image, the demineralised lesion appears as a cluster of increased brightness with decreased reflectivity just beneath the lesion.^3, 18^ As demineralisation progresses, minerals are lost completely and enamel starts to degenerate resulting in appearance of a dark cavitated lesion.^26^ Moreover, fissure sealants FJ and BT appeared to be worn in the OCT images of successive weeks, showing surface irregularities with increased signal reflection throughout the body of the sealant (Figs 2b, 2c, 3b and 3c). This could be related to the effect of acidic medium on the integrity of the material.^12^ While mechanical properties of glass-ionomer are known to be inferior to those of resin composite, the surface microhardness of giomers was also shown to be more affected by acids than composite.^19, 28^

The results of this study demonstrated that S-PRG fillers containing resin sealant and glass-ionomer-based fissure sealant inhibited demineralisation of adjacent enamel significantly greater than conventional resin sealant. HL sealant lacked the inhibitory effect and the neutralising ability of glass-ionomer and giomers,^13^ resulting in the formation of deep lesions that progressed rapidly adjacent to the sealant margins. Moreover, the demineralisation progress was reported to be influenced by marginal seal of the restoration.^26^ For resin composite, the marginal seal is compromised by the inherent property of polymerisation shrinkage, inorganic filler content and surface treatment.^2^ Therefore, it was speculated that the presence of interfacial gaps increased the susceptibility of enamel adjacent to HL to demineralisation.^20^

The statistically significant reduction in the size of lesions in BT and FJ together with slower progression of demineralisation was attributed to ions release from fluoroaluminosilicate and S-PRG fillers. Both fillers have relatively high fluoride content and release abundant fluoride to the local environment.^9^ Moreover, previous studies have shown that fluoride release from glass-ionomer is enhanced in acidic medium.^4^ The presence of fluoride plays an essential role in reducing the incidence and progression of demineralisation at enamel-sealant interface.^13^ Furthermore, incorporation of the released fluoride into the demineralised lesion may promote remineralisation and improve demineralisation resistance.^13^ In addition, other ions might be involved in acid buffering and retardation of enamel demineralisation. Strontium ion released from S-PRG fillers increases the acid resistance of enamel and has a synergistic effect with fluoride in replacing calcium and hydroxyl ions in enamel apatite.^13, 23^ This may justify the presence of acid resistant zones in the OCT images of BT and FJ. Nevertheless, these zones were diminished gradually and subsurface lesions were subsequently formed at the sealant margin after 4 weeks.

In this study, a greater reduction in the mean lesion size of FJ was observed when compared with BT. In addition, the demineralisation inhibition zones were more frequently observed with FJ. This difference may be attributed to the increased content and release of fluoride from FJ.^9^ It has been previously reported that giomers have lower cumulative fluoride release than glass-ionomer, which was proposed to be related to the structure, setting mechanism and presence of silane coupling in the pre-reacted S-PRG fillers.^9, 23^ With a lower concentration of available fluoride, a reduced enamel fluoride uptake and less evident inhibition effect would be anticipated.

In conclusion, the tested null hypotheses were rejected as sealants were different in lesion size and demineralisation progression rate. While conventional resin sealants perform by isolating the fissure, ion-releasing sealants offer additional protective effect. Inhibitory ions would not only benefit the fissure, but also protect adjacent tooth structure against demineralization.
